# Analysis of RNA Interference Lines Identifies New Functions of Maternally-Expressed Genes Involved in Embryonic Patterning in *Drosophila melanogaster*

**DOI:** 10.1534/g3.115.017517

**Published:** 2015-03-31

**Authors:** Niankun Liu, Paul Lasko

**Affiliations:** Department of Biology, McGill University, 3649 Promenade Sir William Osler, Montréal, Québec, Canada H3G 0B1

**Keywords:** embryonic patterning, germ plasm, localized mRNAs, oogenesis, pole cells

## Abstract

Embryonic patterning in *Drosophila melanogaster* is initially established through the activity of a number of maternally expressed genes that are expressed during oogenesis. mRNAs from some of these genes accumulate in the posterior pole plasm of the oocyte and early embryo and localize further into RNA islands, which are transient ring-like structures that form around the nuclei of future primordial germ cells (pole cells) at stage 3 of embryogenesis. As mRNAs from several genes with known functions in anterior–posterior patterning and/or germ cell specification accumulate in RNA islands, we hypothesized that some other mRNAs that localize in this manner might also function in these developmental processes. To test this, we investigated the developmental functions of 51 genes whose mRNAs accumulate in RNA islands by abrogating their activity in the female germline using RNA interference. This analysis revealed requirements for *ttk*, *pbl*, *Hip14*, *eIF5*, *eIF4G*, and *CG9977* for progression through early oogenesis. We observed dorsal appendage defects in a proportion of eggs produced by females expressing double-stranded RNA targeting *Mkrn1* or *jvl*, implicating these two genes in dorsal–ventral patterning. In addition, posterior patterning defects and a reduction in pole cell number were seen in the progeny of *Mkrn1* females. Because the mammalian ortholog of Mkrn1 acts as an E3 ubiquitin ligase, these results suggest an additional link between protein ubiquitination and pole plasm activity.

mRNA localization to particular intracellular regions is widespread. In the early *Drosophila* embryo, mRNA localization, coupled to spatially dependent translational regulation, contributes to targeting the proteins and the localized mRNAs encode to the region of the embryo that is appropriate for their developmental function (Lécuyer *et al.* 2007; Kugler and Lasko 2009). Hundreds of mRNAs have been identified that accumulate in the posterior pole plasm of the early *Drosophila* embryo, where cytoplasmic determinants specify the germ line (Lécuyer *et al.* 2007; Fisher *et al.* 2012). Although a great deal has been learned about how several of these mRNAs function in embryonic patterning and specifying the germ line, for the majority little is known about what role, if any, they have. Several maternal mRNAs that are essential for establishment of the anterior–posterior pattern and for specification of germ cells, including *aret*, *exu*, *gcl*, *nos*, *orb*, *pgc*, and *spir*, are among approximately 50 known mRNAs that transiently accumulate in rings, sometimes termed “RNA islands,” that become apparent around the pole cell nuclei just prior to completion of their cellularization (Lécuyer *et al.* 2007, images publicly available at http://fly-fish.ccbr.utoronto.ca). This suggests a fundamental role for these perinuclear structures, and their constituent mRNAs, in embryonic patterning and germ cell specification. However, the functions of most mRNAs that localize to these structures in pattern formation or germ cell specification are unknown, because mutations affecting them are lethal, or because mutations block oogenesis before mature eggs that can be fertilized are formed, or because no mutants are available.

To address germline-specific functions of essential genes, genetic approaches have been developed to abrogate the functions of specific genes only in germline cells. One such approach involves inducing mitotic recombination and selecting for recombinants using a chromosome carrying a dominant female sterile mutation (Perrimon and Gans 1983). This technique has been used to screen for maternal functions of many zygotically essential genes (Perrimon *et al.* 1984; Perrimon *et al.* 1989); however, it is laborious and such screens have yet to be extended to the entire genome. A more recent approach to this problem is based on the principle of RNA interference (RNAi), in which expression of a small double-stranded hairpin RNA (shRNA) including sequences homologous to a target mRNA post-transcriptionally inactivates the target through translational repression and degradation (Fire *et al.* 1998). Publicly accessible libraries of *Drosophila* lines that express hairpin RNA targeting most protein-coding genes under the control of the upstream activation sequence (UAS) have been assembled (Mummery-Widmer *et al.* 2009; Ni *et al.* 2011). With the use of the appropriate GAL4 driver, these enable, in principle, the specific inactivation of nearly any gene in any tissue, including germline.

To investigate potential functions of mRNAs that accumulate in RNA islands in embryonic patterning or germ cell specification, in this work we conducted a comprehensive analysis of the phenotypes that result during oogenesis or in progeny embryos from maternal germline-specific expression of shRNA that targets each mRNA that accumulates in these perinuclear structures.

## Materials and Methods

### *Drosophila* strains

shRNA-expressing stocks were obtained from the Bloomington stock center. Stock numbers are shown in Table 1. The full genotypes of all the lines used in this study are available on the TRiP website (http://www.flyrnai.org/TRiP-HOME.html). We used the maternal triple driver *MTD-Gal4* to induce expression of shRNA in germ line cells throughout oogenesis (Petrella *et al.* 2007), and we obtained this stock from the Bloomington stock center (stock number 31777).

**Table 1:** Summary of visible phenotypes of RNAi knockdown lines

Gene Name	RNAi KD at 25°	RNAi KD at 29°	Hatch Rate at 25°	Hatch Rate at 29°	Egg Laying	Cuticle Defect	Pole Cell Formation Defect	Pole Cell Migration Defect	Dorsal Appendage Defect	Bloomington Stock Number
*wt*	−	−	93%	84%	Yes	−	−	−	−	
*Ack*	++++		>80%		Yes	−	−	−	−	35264
*Ank*	++	+++	>80%	67%	Yes	−	−	−	−	43965
*aret*					No					35394
*Bsg25D*	−	−	>80%	>80%	Yes	−	−	−	−	36828
*CAH2*	−	++++	>80%	62%	Yes	+	−	−	−	41836
*CG10077*	++	++++	>80%	73%	Yes	−	−	−	−	32388
*CG11597*	++	++	>80%	>80%	Yes	−	−	−	−	43175
*CG14322**^a^*										N/A
*CG18446*	−	−	>80%	78%	Yes	−	−	−	−	33735
*CG2865*	++	++	>80%	71%	Yes	−	−	−	−	43165
*CG31998*	−	+++	>80%	12%	Yes	++	++	−	−	41828
*CG3295**^a^*										N/A
*CG4040*	++++		0%		Yes	+++	+++		−	42776
*CG5292*	−	+++	>80%	>80%	Yes	−	−	−	−	32499
*CG6509*	−	++++	>80%	78%	Yes	−	−	−	−	41832
*CG9821*	+++	++++	68%	55%	Yes	++	−	−	−	43171
*CG9977*					No					43168
*Charybde*	−	+	>80%	>80%	Yes	−	−	−	−	43975
*Cta*	+	+	5%	0%	Yes	+++	−	+++	−	41964
*CycB*	−	++++	>80%	>80%	Yes	−	−	−	−	39024
*Del*	++++		0%		Yes	+++	+++		−	32375
*Dock*	++	++++	>80%	77%	Yes	−	−	−	−	43176
*eIF-4G*					No					33049
*eIF5*					No					34841
*Exu*	++++		0%		Yes	+++	+++		−	41816
*Gap1*	++++		>80%		Yes	−	−	−	−	41830
*Gcl*	−	++++	>80%	>80%	Yes	−	−	−	−	34608
*Gwl*	++++		0%		Yes	+++	+++		−	35212
*Hip14*					No					35012
*Jvl*	+++	+++	78%	36%	Yes	++	−	−	++	43177
*mei-P26*					No					36855
*Milt*	++++		41%		Yes	++	−	++	−	43173
*Mkrn1*	+++	++++	76%	78%	Yes	++	++	−	++	43178
*Nos*	+	+++	1.5%	0%	Yes	+++	++		−	33973
*nrv1*	++++		0%		Yes	+++	+++		−	41829
*Orb*					No					43143
*Osk*	++++		0%		Yes	+++	+++		+++	36903
*pAbp*	−	−	>80%	69%	Yes	−	−	−	−	36127
*Patr-1*	++	+++	>80%	47%	Yes	+	−	−	−	34667
*Pbl*					No					36841
*Pgc*	−	−	>80%	>80%	Yes	−	+	−	−	33720
*Pino*	+	+	>80%	>80%	Yes	−	−	−	−	43971
*Pi3K21B*	++	+++	>80%	59%	Yes	−	−	−	−	36810
*Pum*	++	++++	>80%	>80%	Yes	−	−	−	−	41875
*Rapgap1*	++	+++	72%	48%	Yes	++	++	−	−	42782
*Sl*	+++	++	>80%	78%	Yes	−	−	−	−	35604
*Spir*	++	++	21%	8%	Yes	+++	+++		−	43161
*Sra*	+	++++	>80%	66%	Yes	−	−	−	−	36900
*Tao*	++++		0%		Yes	+++	−	++	−	35147
*Tm1*	+++	++++	>80%	>80%	Yes	−	−	−	−	38232
*Ttk*	−		>80%		No (29°)					36748
*Unr*	+++	++++	>80%	>80%	Yes	−	−	−	−	32432
*Vas*	++++		0%		Yes	+++	+++		−	38924

a RNAi stock not available.

### Screen setup

Ten to 15 *MTD-Gal4* males were crossed to 10–15 virgin females of each TRiP line in a vial and transferred to fresh food every 3–5 d. Crosses were incubated at 25° throughout the experiment or, alternatively, the flies were discarded after 5 d and the vials containing larvae were transferred to 29° to complete development. Growth of females with MTD-Gal4-driven shRNAs at 29° sometimes produces more severe phenotypes and more effective knockdown of the target mRNA (Ni *et al.* 2011; this study). Progeny carrying both *MTD-Gal4* and the shRNA construct were collected from these crosses, eggs were collected, and their phenotypes were assessed as described below.

### Cuticle preparation, hatch rate determination, and dorsal appendage preparation

Cuticle preparations were performed as described in Nüsslein-Volhard *et al.* (1984) with the following modifications: 30–50 flies, of both sexes in approximately equal proportions, were transferred into egg-laying cages with apple juice agar plates (60-mm × 15-mm cell culture dish) supplemented with fresh yeast paste and incubated at 25° or 29°. Genotypes for these crosses are described in *Results*. Collections from the first 2 d after transfer were discarded. Subsequently, eggs were collected either overnight or for 6 hr and were allowed to develop for an additional 36 hr at 25°. Hatch rate was determined by counting the number of hatched eggs and unhatched eggs for each lay. In cases where more than 20% of the eggs failed to hatch, eggs were collected for cuticle preparation as follows: first, embryos were transferred into small sieves and washed with water, then they were dechorionated in a 50% dilution of commercial bleach (12% sodium hypochlorite) for 2 min, and, finally, they were washed with water for another 2 min. The embryos were then transferred with a fine brush into a 1.5-ml microcentrifuge tube containing PBST buffer (1× PBS, and 0.1% Tween-20). Buffer was removed as completely as possible with a micropipette tip, and then 30 µl Hoyer’s medium (30 g gum arabic, 50 ml H_2_O, 200 g chloral hydrate, 20 g glycerol) was added. The embryos were then mounted onto a glass slide and covered with a 22- × 22-mm cover slip. Next, the embryos were cleared by overnight incubation at 65° and observed under dark field illumination using a Leica DM 6000B microscope. To assess dorsal appendage phenotypes, newly laid eggs were transferred onto a glass slide containing PBST buffer and examined under dark field illumination using a Leica DM 6000B microscope.

### Immunohistochemistry

Ovaries were dissected from 3- to 5-d-old females in PBS and fixed in 100 μl of PBS, 1% NP-40, 600 μl of heptane, and 100 μl of 10% formaldehyde for 20 min. Samples were rinsed three times, washed three times for 10 min with PBST (PBS + 0.3% Triton X-100), and blocked in PBSTA (PBST + 1% BSA) for 1 hr at room temperature. Samples were incubated with primary antibodies overnight at 4° in PBSTA. Samples were rinsed three times, washed three times (20 min each) with PBST, and then blocked in 1 ml of PBSTA for 1 hr at room temperature. Samples were incubated in the dark with fluorescent secondary antibody (pre-adsorbed goat anti-rat Alexa Fluor 488 and goat anti-rabbit Alexa Fluor 555; Life Technologies), final dilution 1:1000 in PBSTA overnight at 4°, then washed four times (5 min each) and twice (15 min each) in PBST in the dark. Samples were counterstained with DAPI, mounted in anti-fade reagent in glycerol/PBS from the SlowFade Antifade Kit (Molecular Probes), and examined under a confocal microscope (Zeiss LSM510). Rat anti-Vas was used at a dilution of 1:1000. Rabbit anti-Osk was used at a dilution of 1:1000. Embryos were immunostained as described in Kobayashi *et al.* (1999). Primary rabbit anti-Vas was used at 1:5000. Secondary antibody was anti-rabbit Alexa Fluor 488 (Life Technologies). Images were collected on a Leica DM 6000B microscope.

### RNA isolation and RT-PCR analysis

Total RNA was prepared from 30 embryos (0–2 hr at 25° or 0–1 hr at 29°) using TRIzol reagent (Life Technologies) according to the manufacturer’s protocol, followed by treatment with TURBO DNase (Ambion) for 30 min at 37°. First strand cDNA was synthesized with Maxima H Minus First Strand cDNA Synthesis Kit (Thermo Scientific). Quantitative differences in gene expression were determined by PCR with JumpStart REDTaq ReadyMix Reaction Mix (Sigma) using the first strand cDNA as a template. PCR products (5 μL for each) were resolved on a 1.5% agarose gel using primers that produced a product between 150 and 250 bp in length. Primers that amplify *rp49* mRNA served as a positive control.

## Results

Our results are described below and are summarized in tabular form (Table 1).

### Maternal-effect loci involved in embryonic patterning

**Figure 1:**
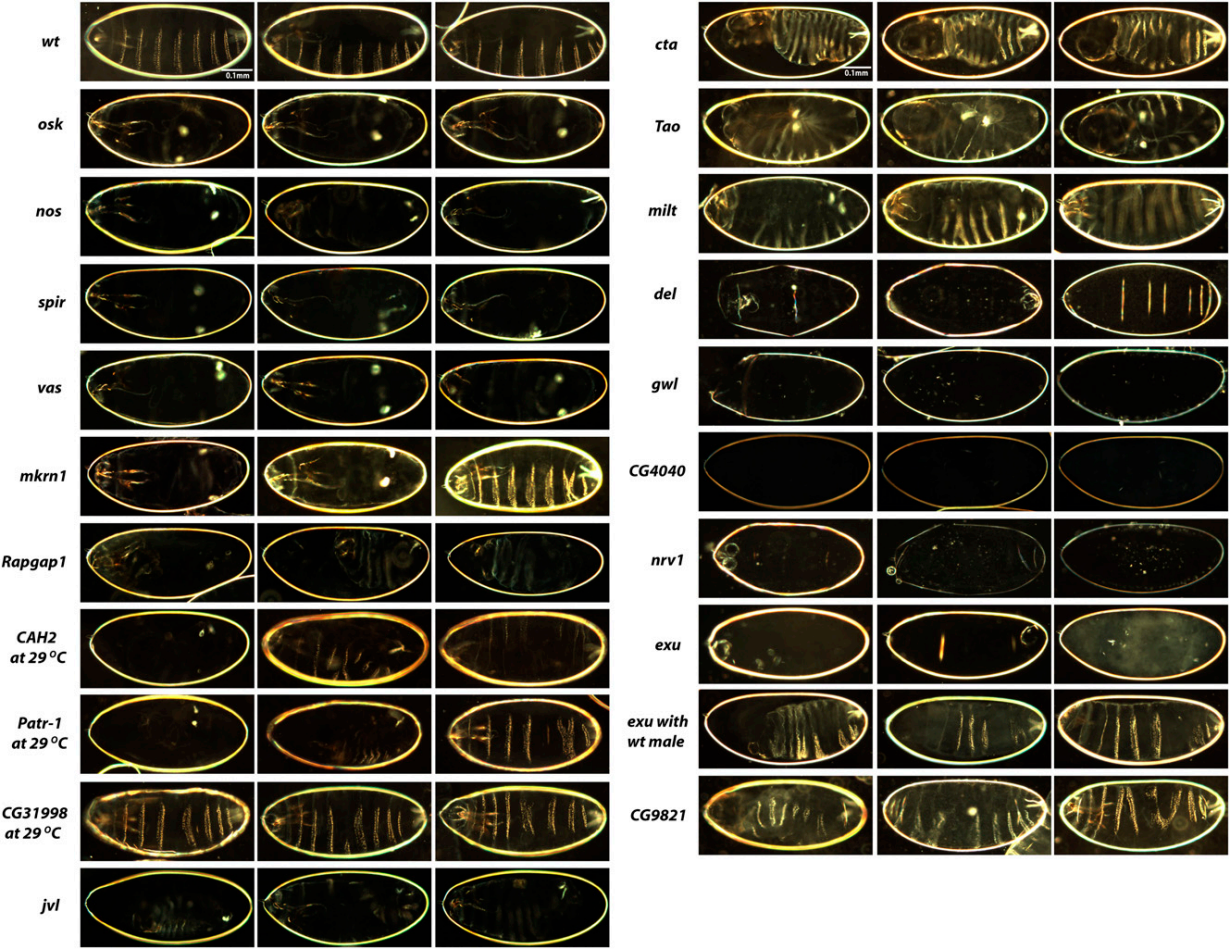
Dark-field photographs of cuticle preparations of RNAi knockdown embryos. Three embryos are illustrated from each knockdown line to capture the range of phenotypic severity that was observed. Embryos are oriented with anterior to the left. Control wild-type (wt) embryos are shown in the top row. The phenotypes observed for each line are discussed in *Results*.

We examined cuticle preparations from embryos produced by females expressing each RNA interference construct (henceforth referred to as knockdown embryos for brevity) that failed to hatch into larvae as a first step in characterizing their phenotype. In wild-type embryos that are about to hatch, the most prominent anterior structures are the mouth parts, which protrude from the anterior pole into the interior of the embryo (Figure 1, **wt**, seen most easily in the leftmost panel). Three thoracic segments and eight abdominal segments are then marked by transverse bands of short bristles called denticle belts; these are very fine and narrow for the three thoracic segments, but are broader and more prominent for the eight abdominal segments (Figure 1, **wt**). At the most posterior structure is a pair of structures, collectively termed the telson (Figure 1, **wt**, seen most easily in the rightmost panel).

Consistent with known phenotypes for the corresponding mutants (Lehmann and Nüsslein-Volhard 1986; Schüpbach and Wieschaus 1986; Nüsslein-Volhard *et al.* 1987; Manseau and Schüpbach 1989), most *osk*, *nos*, *spir*, and *vas* knockdown embryos exhibited a strong grandchildless-*knirps* phenotype (Schüpbach and Wieschaus 1986). These embryos are shorter than wild-type, lack most posterior segmentation, and have two prominent foci of telson-derived material, surrounded by mostly naked cuticle (Figure 1, ***osk*, *nos*, *spir*, *vas***). A substantial proportion (∼25%) of *nos* embryos cultured at 29° completely failed to develop and did not form cuticles. Interestingly, we discovered a similar grandchildless-*knirps* phenotype in 5–10% of *mkrn1* knockdown embryos that failed to hatch (Figure 1, ***mkrn1***, left and center panels), although posterior defects were less extreme in some of these embryos with most posterior denticle belts apparent (Figure 1, ***mkrn1***, right panel). Severe posterior patterning defects were also observed in some *Rapgap1*, *CAH2*, *Patr-1*, and *jvl* embryos (Figure 1, ***Rapgap1*, *CAH2*, *Patr-1*, *jvl***). These embryos differed from grandchildless-*knirps* embryos, however, in that most did not completely fill the entire volume of the egg and appeared shriveled, presumably as a result of holes in their cuticles. For *CAH2* and *Patr-1*, these phenotypes were incompletely penetrant and many embryos appeared normal, whereas for *Rapgap1* and *jvl* most embryos were affected. *CG31998* knockdown embryos also exhibited defects in anterior–posterior patterning, but to a lesser degree than for those previously mentioned. In some *CG31998* knockdown embryos, the fourth abdominal segment was partially or fully absent, or fused with the fifth (Figure 1, ***CG31998***).

**Figure 2:**
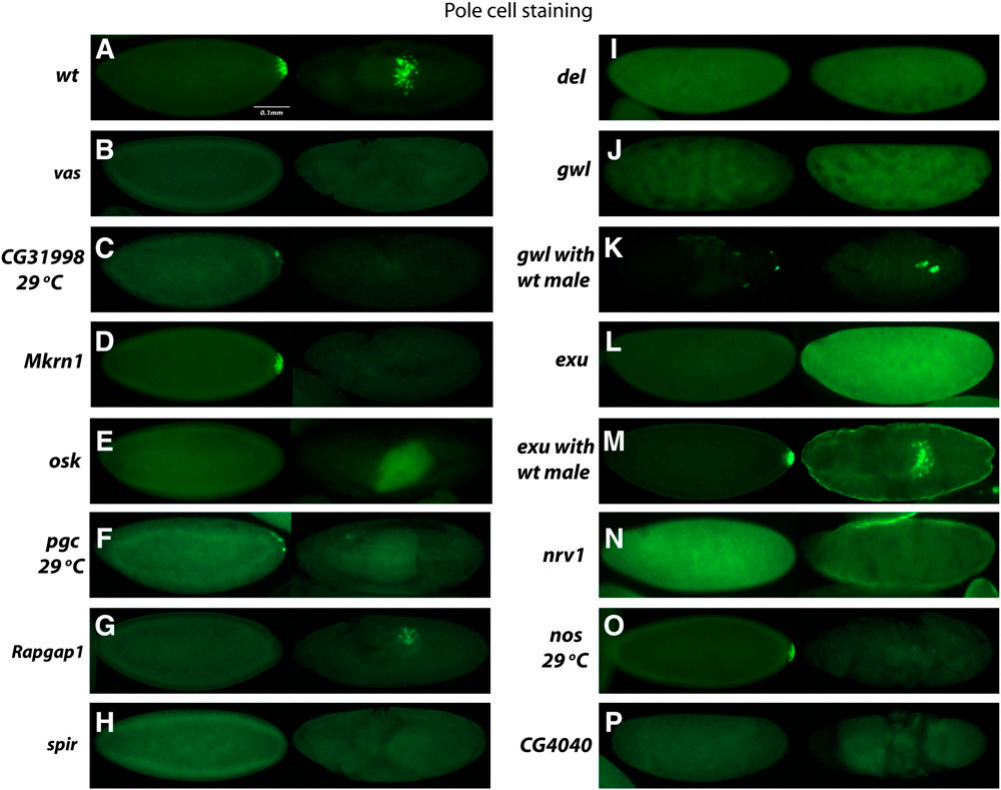
Embryos derived from RNAi knockdown mothers were stained for Vas protein (green) to visualize pole cells. Two embryos are shown for each knockdown line. For those that develop sufficiently, the embryo in the left panel is at the blastoderm stage, whereas the embryo in the right panel is at stage 10, which is the stage at which pole cells are in mid-migration or later. In many cases development does not progress normally beyond the blastoderm stage, and in these instances the embryo in the right panel represents what appears to be the latest stage of development achieved. In some cases, development ceases before cellularization, and then two representative embryos are shown. Wild-type embryos (wt), for comparison, are shown in the first row. The phenotypes observed for each line are discussed in *Results*.

Consistent with the known phenotype for the corresponding mutant (Schüpbach and Wieschaus 1989), *cta* knockdown embryos failed to properly complete gastrulation. The embryos form a twisted structure with anterior holes (Figure 1, ***cta***). *Tao* knockdown embryos progress through germ band extension but then do not retract, so they form U-shaped cuticles (Figure 1, ***Tao***). These embryos also have obvious head defects. In *milt* knockdown embryos, various segments are partially missing or are fused and telsons are also often missing or reduced to rudiments (Figure 1, ***milt***). *del*, *gwl*, *CG4040*, *nrv1*, and *exu* knockdown embryos do not progress sufficiently in development to form cuticles (Figure 1, ***del*, *gwl*, *CG4040*, *nrv1*, *exu***); however, for *exu* and *gwl* (Figure 2), this phenotype is somewhat suppressed by a wild-type paternal copy of the gene in that cuticles form but severe anterior–posterior patterning defects are apparent, including a loss of anterior structures (Figure 1, ***exu with wt male***). Loss of anterior structures has been reported as a maternal-effect phenotype of *exu* mutations (Schüpbach and Wieschaus 1989), and failure of oocytes to arrest in metaphase I of meiosis, resulting in a failure to support embryogenesis, is a phenotype of a hypomorphic *gwl* allele (Archambault *et al.* 2007). Finally, in many *CG9821* knockdown embryos, mouth parts are malformed and there is loss or fusion of abdominal segments (Figure 1, ***CG9821***). Other *CG9821* knockdown embryos are, however, patterned normally.

### Examination of pole cells in knockdown embryos with defects in embryonic development and assessment of dorsal appendages

**Figure 3:**
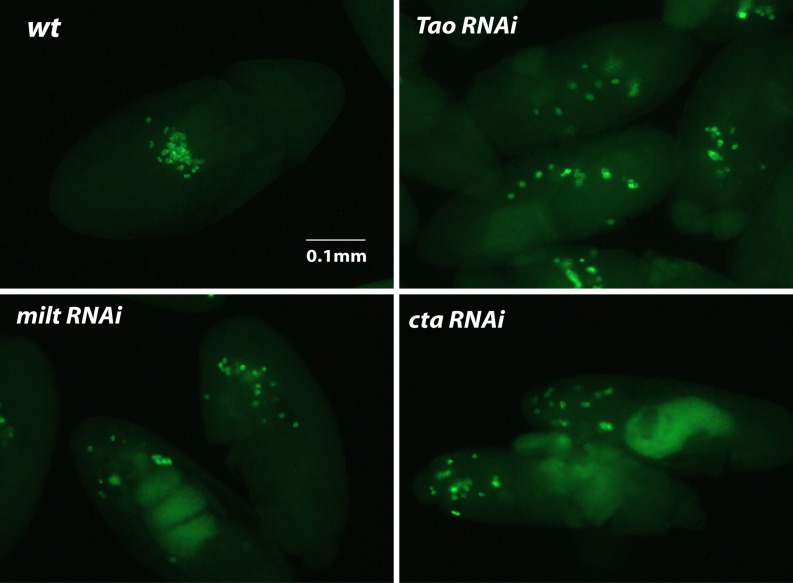
Embryos derived from RNAi knockdown mothers were stained for Vas protein (green) to visualize pole cell migration defects. Wild-type embryos, for comparison, are shown in the first picture. The phenotypes observed for each line are discussed in *Results*.

**Figure 4:**
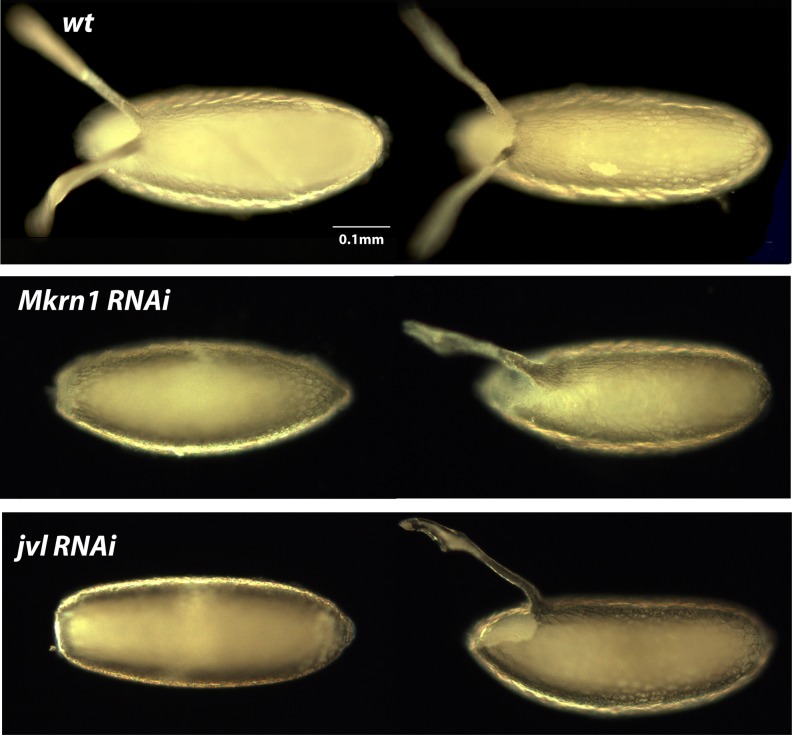
Dark-field photographs of dorsal appendage defects of RNAi knockdown embryos. Two embryos are illustrated from each knockdown line to capture the range of phenotypic severity that was observed. Wild-type embryos (wt), for comparison, are shown in the first row. The phenotypes observed for each line are discussed in *Results*.

Next, we examined these knockdown embryos for their ability to form pole cells by immunostaining with anti-Vas (Figure 2). In wild-type, pole cells form at the posterior pole prior to general cellularization (Figure 2A, left panel). At gastrulation, they migrate along with the posterior midgut invagination into the interior of the embryo and then migrate as individual cells (Figure 2A, right panel) until forming two clusters in association with the gonadal mesoderm to form the two gonads. Knockdown embryos for known posterior-group genes (*vas*, *osk*, *spir*; Figure 2, B, E, and H) and those that did not form cuticles (*del*, *gwl*, *exu*, *nrv1*, *CG4040*; Figure 2, I, J, L, N, and P) also did not form pole cells, although this phenotype was completely rescued for *exu* (Figure 2M) and partially rescued for *gwl* (Figure 2K) by a paternal wild-type copy of the gene . In this case, approximately 50% of *gwl* knockdown embryos formed pole cells in numbers smaller than those for wild-type. Consistent with the phenotype of the corresponding mutant, and that of embryos produced by females expressing antisense RNA targeting *pgc* (Nakamura *et al.* 1996; Martinho *et al.* 2004), we observed a severe reduction in pole cell number in *pgc* knockdown embryos; pole cells were absent in 20% of embryos and present in reduced numbers in the remaining 80% (Figure 2F). For *CG31998*, 15% of knockdown embryos formed 0–5 pole cells and the rest formed wild-type numbers of pole cells (Figure 2C). A similar phenotype was observed for *Mkrn1*, with approximately 20% of embryos forming 0–5 pole cells (Figure 2D). Pole cells were also absent in approximately 25% of *Rapgap1* embryos (Figure 2G). For *nos* knockdown embryos cultured at 29°, pole cells formed in normal numbers and were localized normally until the onset of pole cell migration (Figure 2O, left panel). In later-stage embryos, pole cell migration was highly aberrant and pole cell numbers diminished as development progressed, such that stage 14 and later embryos had only a few scattered pole cells (Figure 2O, right panel) or none at all. In knockdown embryos for *Tao*, *milt*, and *cta*, wild-type numbers of pole cells form, but they also frequently scatter during migration. This is presumably because of the extensive somatic defects that are present in these embryos. We observed failure of pole cells to coalesce into gonads in 53% of *Tao* knockdown embryos, 36% of *milt* knockdown embryos, and 97% of *cta* knockdown embryos (Figure 3). Our results differ from observations of embryos produced by a *Tao* hypomorphic mutant where reduced numbers of pole cells were present (Sato *et al.* 2007). We also observed defects in dorsal appendage structure in eggs produced by *Mkrn1* and *jvl* knockdown females (Figure 4). For *Mkrn1* knockdowns, 15% of eggs lacked dorsal appendages and 18% had a single fused dorsal appendage, whereas for *jvl* knockdowns 19% of eggs lacked dorsal appendages and 24% had a single fused dorsal appendage. Similar dorsal appendage defects have been reported in eggs produced from a hypomorphic *jvl* mutant (Dubin-Bar *et al.* 2011).

### Knockdown of some genes blocked oogenesis

**Figure 5:**
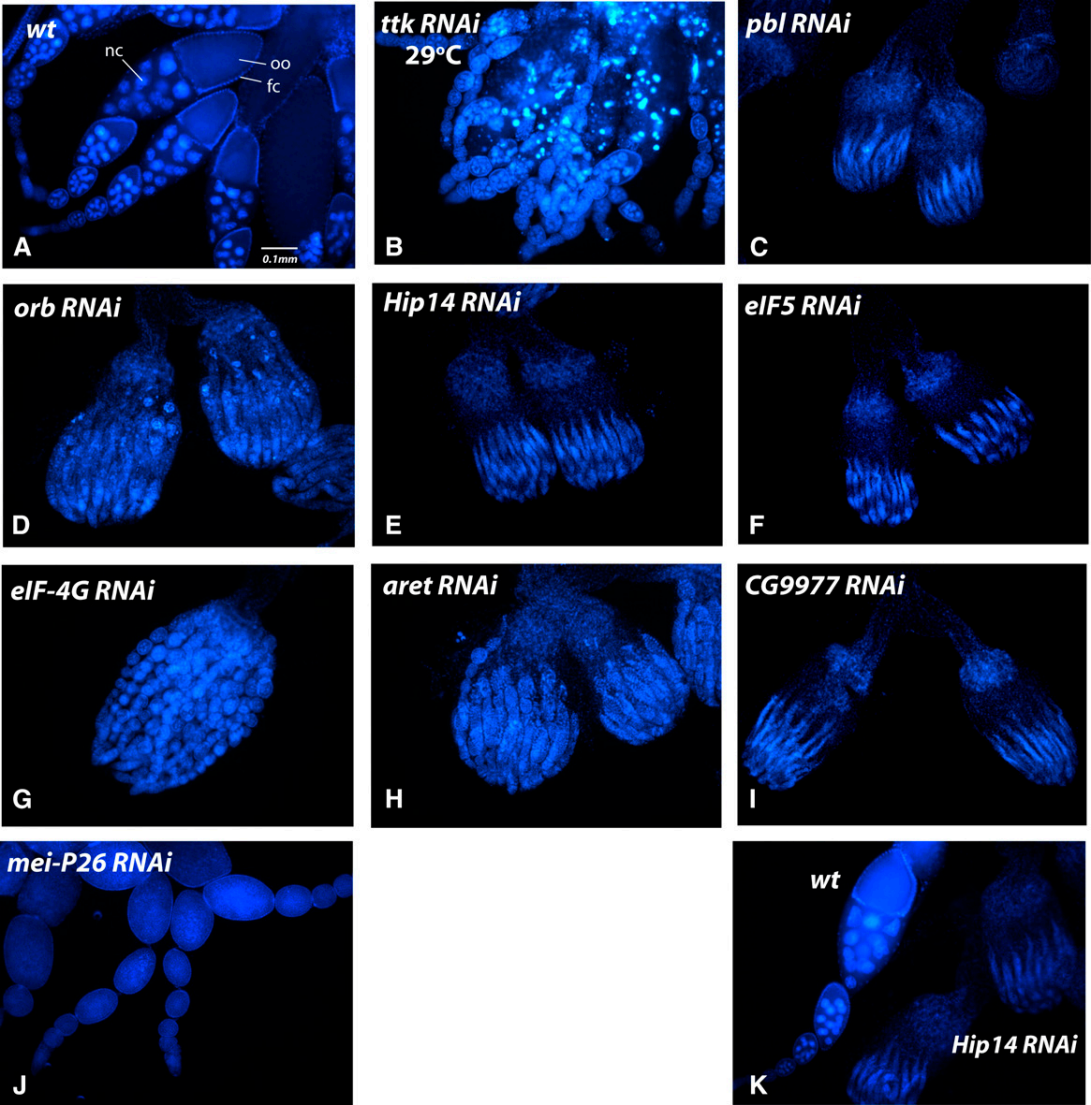
Ovaries derived from RNAi knockdown mothers that did not lay eggs were visualized by DAPI staining (blue). Wild-type ovaries, for comparison, are shown in the first picture, and the oocyte (oo), 15 nurse cells (nc), and follicle cells (fc) are labeled. The phenotypes observed for each line are discussed in *Results*. In the bottom right panel, a single wild-type ovariole and two entire ovaries from the *Hip14* shRNA expressing line are photographed together to illustrate the difference in size and extent of development.

Knockdown of another set of genes whose mRNAs accumulate in pole cell rings resulted in defects during oogenesis that prevented the development of mature eggs. In these cases, we analyzed the morphology of the ovaries that were produced (Figure 5). The earliest developmental blocks in oogenesis occurred in females knocked down for *pbl* (Figure 5C), *Hip14* (Figure 5E), *eIF5* (Figure 5F), or *CG9977* (Figure 5I). In these cases, essentially no germ line cells were observed, indicating that abrogation of function of these genes results in cellular lethality. Knockdown of *orb* (Figure 5D), *eIF-4G* (Figure 5G), or *aret* (Figure 5H) resulted in the formation of some rudimentary egg chambers, but these did not progress beyond early pre-vitellogenic stages. The phenotypes of *orb* and *aret* knockdowns are consistent with those of known severe mutations in these genes (Schüpbach and Wieschaus 1991; Christerson and McKearin 1994). Knockdown of *mei-P26* led to the formation of tumorous egg chambers similar to those described in *mei-P26* mutants (Figure 5J) (Page *et al.* 2000). Knockdown of *ttk* resulted in normal oogenesis until approximately stage 6, followed by extensive cell death (Figure 5B).

### Assessment of efficacy of RNAi knockdown

**Figure 6:**
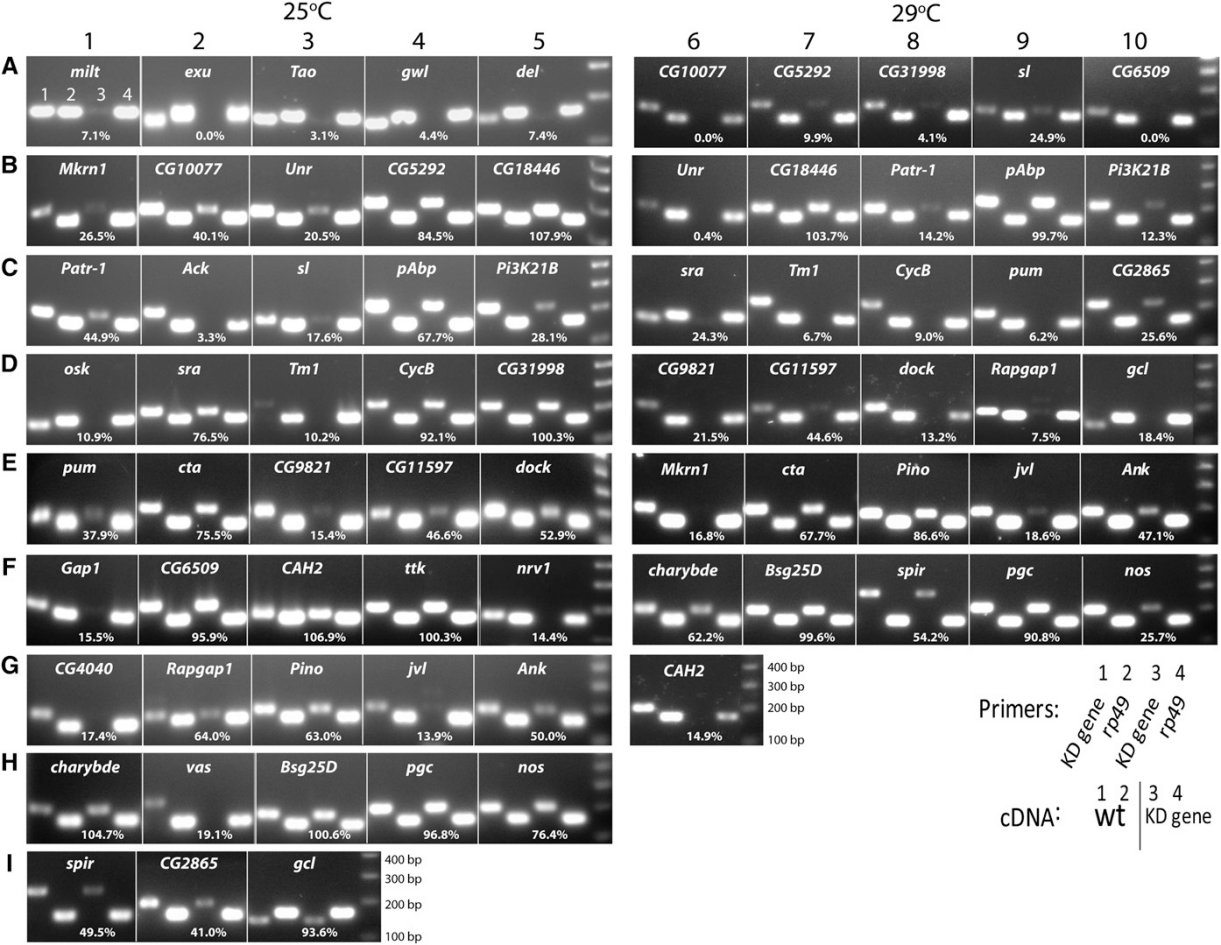
Analysis of the efficacy of knockdown of each gene by RT-PCR analysis. The name of the targeted gene and four lanes of gel are shown in each small picture. In each picture, cDNA prepared from wild-type embryos were added in lanes 1 and 2 and cDNA prepared from RNAi knockdown embryos were added in lanes 3 and 4. Primers amplifying the indicated gene were used in lanes 1 and 3 to compare cDNA level in wild-type and knockdown lines. Primers amplifying a control gene knockdown and primers of rp49 were used in lanes 2 and 4. Knockdown is most efficient when the band in lane 3 is absent or very much weaker than the band in lane 1, whereas the bands in lanes 2 and 4 are equally intense. The percentage value in each panel reports the following ratio of band intensities: (lane 3/lane 1) / (lane 4/lane 2). This value measures the efficiency of the knockdown when controlled for potential differences in the amount of RNA used for the PCR reaction in the control and knockdown lanes. 0% represents a total knockdown and 100% represents a completely ineffective knockdown. Band intensities were quantitated using ImageJ software.

We examined the effectiveness of each RNAi construct at targeting its corresponding mRNA using RT-PCR (Figure 6). In total, we attempted to knock down the germline activities of 51 different genes that express mRNAs that localize in perinuclear rings in the precursors to pole cells. For seven of these genes [*Bsg25D* (Figure 6F7), *CG18446* (Figure 6B7), *charybde* (Figure 6F6), *cta* (Figure 6E7), *pAbp* (Figure 6B9), *pgc* (Figure 6F9), *Pino* (Figure 6E8)], the effectiveness of the knockdowns appeared very poor (<40% reduction) by this assay, even when flies were cultured at elevated temperature. Although we did not observe any effects on oogenesis or embryonic viability from expressing shRNA targeting *Bsg25D*, *CG18446*, *charybde*, *pAbp*, or *Pino*, we cannot conclude that these genes play no essential role in the female germ line because targeting them in this way was inefficient. Surprisingly, despite apparently poor efficiency of the corresponding shRNA, we nevertheless obtained a developmental phenotype for *cta* and *pgc*, as described in previous sections. For *ttk*, knockdown was poor when shRNA-expressing flies were cultured at 25° (Figure 6F4) and no phenotype resulted, but culture at 29° resulted in an early block in oogenesis (Figure 5), presumably implying effective knockdown but also making it impossible to collect embryos for RT-PCR analysis.

Conversely, knockdown of 27 genes appeared complete or nearly complete (80–100%) by RT-PCR at one or both temperature conditions. These genes in alphabetical order were *Ack* (Figure 6C2), *CAH2* (Figure 6G6), *CG4040* (Figure 6G1), *CG5292* (Figure 6A7), *CG6509* (Figure 6A10), *CG10077* (Figure 6A6), *CG31998* (Figure 6A8), *CycB* (Figure 6C8), *del* (Figure 6A5), *dock* (Figure 6D8), *exu* (Figure 6A2), *Gap1* (Figure 6F1), *gcl* (Figure 6D10), *gwl* (Figure 6A4), *jvl* (Figure 6E9), *milt* (Figure 6A1), *Mkrn1* (Figure 6E6), *nrv1* (Figure 6F5), *osk* (Figure 6D1), *Patr-1* (Figure 6B8), *PI3K21B* (Figure 6B10), *pum* (Figure 6C9), *Rapgap1* (Figure 6D9), *Tao* (Figure 6A3), *Tm1* (Figure 6D3), *Unr* (Figure 6B6), and *vas* (Figure 6H2). The phenotypes of *MTD-Gal4*-driven expression of shRNAs targeting 15 of these genes (*CAH2*, *CG4040*, *CG31998*, *del*, *exu*, *gwl*, *jvl*, *milt*, *Mkrn1*, *nrv1*, *osk*, *Patr-1*, *Rapgap1*, *Tao*, and *vas*) have been described above. For the other 12 (*Ack*, *CG5292*, *CG6509*, *CG10077*, *CycB*, *dock*, *Gap1*, *gcl*, *Pi3K21B*, *pum*, *Tm1*, and *Unr*), we observed no effect on oogenesis or embryonic development. This is a surprising result for *gcl* and *pum* because the requirements for *gcl* in germline for establishment of the germ cell lineage and of *pum* for posterior patterning and germ cell maintenance are well-established (Asaoka-Taguchi *et al.* 1999; Parisi and Lin 1999; Robertson *et al.* 1999). We also expected to observe phenotypes in *Tm1* knockdown embryos because several *Tm1* mutations virtually abrogate *osk* localization and germline clones of a *Tm1* null allele produce sterile adults or embryos lacking germ cells and abdominal segments (Erdélyi *et al.* 1995). Also, *CycB* mutants are female-sterile and produce rudimentary ovaries (Jacobs *et al.* 1998). We conclude that even in cases where knockdown appears efficient, lack of a phenotype from shRNA expression does not rule out involvement of a particular gene in oogenesis or embryonic patterning.

Our attempts at knockdown of eight other genes were only partially successful (40–80%). These genes, in alphabetical order, were *Ank* (Figure 6E10), *CG2865* (Figure 6C10), *CG9821* (Figure 6D6), *CG11597* (Figure 6D7), *nos* (Figure 6F10), *sl* (Figure 6A9), *spir* (Figure 6F8), and *sra* (Figure 6C6). As discussed above, we nevertheless obtained phenotypes from knockdown of *CG9821*, *nos*, and *spir*, but it remains possible that more severe phenotypes, or phenotypes that manifest earlier, would have been observed if the knockdowns were more efficient. For the remainder of these genes where partial knockdowns did not produce effects on oogenesis or embryogenesis, we cannot draw any conclusions about potential roles for them in these processes.

For the nine genes, including *ttk* at 29°, for which knockdown produced developmental blocks in early stages in oogenesis (Figure 5), we did not analyze the effectiveness of the knockdown in this manner because tissue from their rudimentary ovaries was difficult to obtain and appropriate controls were lacking.

**Figure 7:**
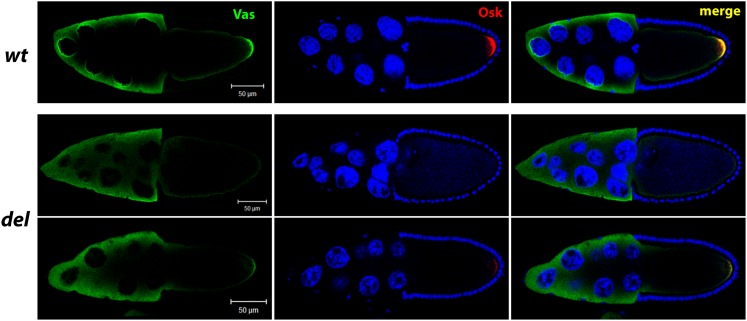
*del* RNAi affects early pole plasm formation. Ovaries derived from RNAi knockdown mothers who lay eggs, but where pole cells do not form, were immunostained for Vas protein (green) and Osk protein (red) to visualize pole plasm formation. Nuclei were visualized by DAPI staining (blue). Wild-type ovaries, for comparison, are shown in the first row. ***del RNAi***: Accumulation of VAS at the oocyte posterior and nuage in nurse cells are reduced while cytoplasmic nurse cell VAS levels are normal. Posterior OSK levels are also reduced.

Finally, we examined whether two pole plasm components, Osk and Vas protein, localized normally to the posterior of the stage-10 oocyte in the knockdown lines where pole cell formation was compromised. In flies expressing shRNA targeting *del*, we found that posterior accumulation of both Osk and Vas was greatly reduced, as was accumulation of Vas into the perinuclear nuage, but that the level of Vas in the cytoplasm of nurse cells was comparable to that of controls (Figure 7). Conversely, for flies expressing shRNA targeting *CG4040*, *exu*, *gwl*, or *nrv1*, Vas and Osk accumulation appeared similar to that of wild-type (data not shown).

## Discussion

In this study we analyzed a set of mRNAs that accumulate in cytoplasmic rings within primordial germ cell precursors, sometimes called “RNA islands,” by expressing in germline shRNAs that target them and examining the phenotypic consequences. This work provided evidence for specific roles in embryonic patterning and/or germline specification for several genes whose mRNAs localize in this way and that have not been previously implicated in these processes. This provides further support for the conclusion that these structures accumulate mRNAs that are involved in these developmental events.

Of particular interest to us are genes that we implicated in anterior–posterior embryonic patterning or in germ cell specification. One of these is *mkrn1*, for which no mutant phenotype had been previously described in *Drosophila*. In mammals, the protein encoded by MKRN1 is an E3 ubiquitin ligase that modifies PPARγ, a transcription factor involved in activating adipocyte differentiation, and targets it for degradation (Kim *et al.* 2014). Several other targets have also been identified for MKRN1, indicating it is involved in numerous cellular and disease-related processes. Previous work has implicated post-translational modification pathways in establishing and maintaining posterior localization of Vas (Liu *et al.* 2003; Kugler *et al.* 2010), and thus in anterior–posterior patterning and pole cell specification. Our present observations raise the possibility that Mkrn1 may regulate the stability by ubiquitinating one or more proteins involved in posterior patterning and pole cell specification.

We also observed posterior embryonic defects in *Patr-1* knockdown embryos. *Patr-1* encodes a component of P bodies that is believed to activate mRNA decapping and miRNA degradation (Jäger and Dorner 2010; Pradhan *et al.* 2012; Barišić-Jäger *et al.* 2013; Nishihara *et al.* 2013), and it has also been identified as a component of the somatic piRNA pathway (Handler *et al.* 2013). It has been demonstrated that Patr-1 interacts with the CCR4 deadenylase at the larval neuromuscular junction (Pradhan *et al.* 2012), but its role in germline development has heretofore been unexplored. Given the well-established importance of decapping and CCR4-mediated deadenylation in post-transcriptional genetic regulation in the female germline (Zaessinger *et al.* 2006; Chicoine *et al.* 2007; Tadros *et al.* 2007; Rouget *et al.* 2010; Igreja and Izaurralde 2011), it is probable that the phenotype we observed in *Patr-1* knockdown embryos results from effects on these processes.

The other RNA knockdown lines that produced maternal-effect anterior–posterior defects were *jvl*, *Rapgap1*, and *CAH2*. *jvl* encodes a microtubule-associated protein, and *jvl* mutant oocytes show defects in localization of *grk*, *bcd*, and *osk* mRNA, as well as disruptions of the cytoskeleton (Dubin-Bar *et al.* 2011). Both the mutant and our knockdown line produced embryos with dorsal appendage defects, confirming a role for *jvl* in the germline in producing these structures. Dorsal appendages are produced by follicle cells in response to activation of the epidermal growth factor receptor (Egfr) by its ligand Grk, which is translated in the oocyte from the localized *grk* mRNA and secreted over a short range (González-Reyes *et al.* 1995; Roth *et al.* 1995). Although we did not analyze *grk* mRNA localization in *jvl* (or *Mkrn1*) knockdown embryos because their dorsal appendage phenotypes were not fully penetrant, effects on *grk* mRNA localization, as observed in the *jvl* mutant (Dubin-Bar *et al.* 2011), could explain the effects we observed in these knockdown embryos on dorsal appendage formation.

Unlike in the corresponding mutant, in *jvl* knockdown embryos we did not observe defects in posterior Osk or Vas localization, possibly because of incomplete inactivation of the *jvl* mRNA. Conversely, although the defect in *osk* localization in the *jvl* mutant would be expected to lead to anterior–posterior defects in progeny embryos, this was not observed because *jvl* mutant eggs do not support embryogenesis beyond initial stages. In this instance, then, the incomplete knockdown (or germline specificity of the knockdown) of the target mRNA allowed the identification of a phenotype that was masked in a strong mutant allele. Another similar instance concerned *del*; *del* mutant alleles block oogenesis at an early stage (Schüpbach and Wieschaus 1991), and Del protein has recently been identified as a component of a complex that is targeted to chromatin at dual-strand piRNA clusters and is required to produce piRNAs from those clusters (Mohn *et al.* 2014). Although analysis of rare escaper eggs that progress more completely through oogenesis indicated a later role for *del* in microtubule-mediated processes including localization of *osk* and *grk* mRNAs (Wehr *et al.* 2006), this later phenotype is much more apparent in the RNA knockdown line that produces substantial numbers of embryos.

It is more difficult to predict potential functions for the other two genes in embryonic patterning or germline specification. *Rapgap1* encodes a GTPase activator involved in intracellular signaling, but a null mutant for this gene has been reported to be viable and fertile, with only minor irregularities in pole cell alignment at embryonic stage 13 (Chen *et al.* 1997). Further work will be necessary to determine whether the phenotype we observed results from a secondary off-target effect of the *Rapgap1* shRNA. *CAH2* is one of two *Drosophila* genes that encode a carbonic anhydrase, an enzyme that catalyzes the reversible hydration of carbon dioxide to bicarbonate (Syrjänen *et al.* 2013). No mutant phenotype has been reported for this gene. It is likely to be functionally redundant with CAH1 in most tissues, but high-throughput data indicate that CAH2 is by far the predominant form of the enzyme that is expressed in ovaries (Graveley *et al.* 2011). A role for glycolytic enzymes in germ cell development has recently been described, indicating that metabolic enzymes can have specific developmental roles (Gao *et al.* 2015).

## References

[bib1] Archambault V. Zhao X. White-Cooper H. Carpenter A. T. Glover D. M., 2007 Mutations in *Drosophila* Greatwall/Scant reveal its roles in mitosis and meiosis and interdependence with Polo kinase. PLoS Genet. 3: e200. 17997611 10.1371/journal.pgen.0030200 PMC2065886

[bib2] Asaoka-Taguchi M. Yamada M. Nakamura A. Hanyu K. Kobayashi S., 1999 Maternal Pumilio acts together with Nanos in germline development in Drosophila embryos. Nat. Cell Biol. 1: 431–437. 10559987 10.1038/15666

[bib3] Barišić-Jäger E. Kręcioch I. Hosiner S. Antic S. Dorner S., 2013 HPat a decapping activator interacting with the miRNA effector complex. PLoS ONE 8: e71680. 23977167 10.1371/journal.pone.0071860 PMC3747071

[bib4] Chen F. Barkett M. Ram K. T. Quintanilla A. Hariharan I. K., 1997 Biological characterization of *Drosophila* Rapgap1, a GTPase activating protein for Rap1. Proc. Natl. Acad. Sci. USA 94: 12485–12490. 9356476 10.1073/pnas.94.23.12485 PMC25011

[bib5] Chicoine J. Benoit P. Gamberi C. Paliouras M. Simonelig M., 2007 Bicaudal-C recruits CCR4-NOT deadenylase to target mRNAs and regulates oogenesis, cytoskeletal organization, and its own expression. Dev. Cell 13: 691–704. 17981137 10.1016/j.devcel.2007.10.002

[bib6] Christerson L. B. McKearin D., 1994 *orb* is required for anteroposterior and dorsoventral patterning during *Drosophila* oogenesis. Genes Dev. 8: 614–628. 7926753 10.1101/gad.8.5.614

[bib7] Dubin-Bar D. Bitan A. Bakhrat A. Amsalem S. Abdu U., 2011 *Drosophila javelin-like* encodes a novel microtubule-associated protein and is required for mRNA localization during oogenesis. Development 138: 4661–4671. 21989913 10.1242/dev.069161

[bib8] Erdélyi M. Michon A.-M. Guichet A. Glotzer J. B. Ephrussi A., 1995 Requirement for Drosophila cytoplasmic tropomyosin in *oskar* mRNA localization. Nature 377: 524–527. 7566149 10.1038/377524a0

[bib9] Fire A. Xu S. Montgomery M. K. Kostas S. A. Driver S. E., 1998 Potent and specific genetic interference by double-stranded RNA in *Caenorhaebditis elegans*. Nature 391: 806–811. 9486653 10.1038/35888

[bib10] Fisher, B., R. Weiszmann, E. Frise, A. Hammonds, P. Tomancak *et al.*, 2012 BDGP in situ homepage. Available at: http://insitu.fruitfly.org. Accessed: April 17, 2015

[bib11] Gao M. Thomson T. C. Creed T. M. Tu S. Loganathan S. N., 2015 Glycolytic enzymes localize to ribonucleoprotein granules in *Drosophila* germ cells, bind Tudor and protect them from transposable elements. EMBO Rep. 16: 379–386. 25600116 10.15252/embr.201439694 PMC4364877

[bib12] González-Reyes A. Elliott H. St Johnston D., 1995 Polarization of both major body axes in *Drosophila* by *gurken-torpedo* signalling. Nature 375: 654–658. 7791898 10.1038/375654a0

[bib13] Graveley B. R. Brooks A. N. Carlson J. W. Duff M. O. Landolin J. M., 2011 The developmental transcriptome of *Drosophila melanogaster*. Nature 471: 473–479. 21179090 10.1038/nature09715 PMC3075879

[bib14] Handler D. Meixner K. Pizka M. Lauss K. Schmied C., 2013 The genetic makeup of the *Drosophila* piRNA pathway. Mol. Cell 50: 762–777. 23665231 10.1016/j.molcel.2013.04.031 PMC3679447

[bib15] Igreja C. Izaurralde E., 2011 CUP promotes deadenylation and inhibits decapping of mRNA targets. Genes Dev. 25: 1955–1967. 21937713 10.1101/gad.17136311 PMC3185967

[bib16] Jacobs H. W. Knoblich J. A. Lehner C. F., 1998 *Drosophila* Cyclin B3 is required for female fertility and is dispensable for mitosis like Cyclin B. Genes Dev. 12: 3741–3751. 9851980 10.1101/gad.12.23.3741 PMC317254

[bib17] Jäger E. Dorner S., 2010 The decapping activator HPat is a novel factor co-purifying with GW182 from *Drosophila* cells. RNA Biol. 7: 381–385. 20458171 10.4161/rna.7.3.12088 PMC3173855

[bib18] Kim J.-H. Park K. W. Lee E.-W. Jang W.-S. Seo J., 2014 Suppression of PPARγ through MKRN1-mediated ubiquitination and degradation prevents adipocyte differentiation. Cell Death Differ. 21: 594–603. 24336050 10.1038/cdd.2013.181 PMC3950322

[bib19] Kobayashi S. Amikura R. Nakamura A. Lasko P. F., 1999 Techniques for analyzing protein and RNA distribution in *Drosophila* ovaries and embryos at structural and ultrastructural resolution, pp. 426–445 in Advances in molecular biology: a comparative methods approach to the study of oocytes and embryos, edited by Richter J. Oxford University Press, Oxford, UK.

[bib20] Kugler J.-M. Lasko P., 2009 Localization, anchoring and translational control of *oskar*, *gurken*, *bicoid*, and *nanos* mRNA during *Drosophila* oogenesis. Fly (Austin) 3: 15–28. 19182536 10.4161/fly.3.1.7751

[bib21] Kugler J.-M. Woo J. S. Oh B. H. Lasko P., 2010 Regulation of *Drosophila* Vasa in vivo through paralogous cullin-RING E3 ligase specificity receptors. Mol. Cell. Biol. 30: 1769–1782. 20123973 10.1128/MCB.01100-09 PMC2838069

[bib22] Lécuyer E. Yoshida H. Parthasarathy N. Alm C. Babak T., 2007 Global analysis of mRNA localization reveals a prominent role in organizing cellular architecture and function. Cell 131: 174–187. 17923096 10.1016/j.cell.2007.08.003

[bib23] Lehmann R. Nüsslein-Volhard C., 1986 Abdominal segmentation, pole cell formation and embryonic polarity require the localized activity of *oskar*, a maternal gene in *Drosophila*. Cell 47: 141–152. 3093084 10.1016/0092-8674(86)90375-2

[bib24] Liu N. Dansereau D. A. Lasko P., 2003 Fat facets interacts with Vasa in the *Drosophila* pole plasm and protects it from degradation. Curr. Biol. 13: 1905–1909. 14588248 10.1016/j.cub.2003.10.026

[bib25] Manseau L. Schüpbach T., 1989 *cappuccino* and *spire*: two unique maternal-effect loci requires for both the anteroposterior and dorsoventral patterns of the *Drosophila* embryo. Genes Dev. 3: 1437–1452. 2514120 10.1101/gad.3.9.1437

[bib26] Martinho R. G. Kunwar P. S. Casanova J. Lehmann R., 2004 A noncoding RNA is required for the repression of RNApolII-dependent transcription in primordial germ cells. Curr. Biol. 14: 159–165. 14738740 10.1016/j.cub.2003.12.036

[bib27] Mohn F. Sienski G. Handler D. Brennecke J., 2014 The Rhino-Deadlock-Cutoff complex licenses noncanonical transcription of dual-strand piRNA clusters in *Drosophila*. Cell 157: 1364–1379. 24906153 10.1016/j.cell.2014.04.031

[bib28] Mummery-Widmer J. L. Yamazaki M. Stoeger T. Novatchkova M. Bhalerao S., 2009 Genome-wide analysis of Notch signalling in *Drosophila melanogaster*. Nature 458: 987–992. 19363474 10.1038/nature07936 PMC2988197

[bib29] Nakamura A. Amikura R. Mukai M. Kobayashi S. Lasko P. F., 1996 Requirement for a noncoding RNA in *Drosophila* polar granules for germ cell establishment. Science 274: 2075–2079. 8953037 10.1126/science.274.5295.2075

[bib30] Ni J.-Q. Zhou R. Czech B. Liu L.-P. Holderbaum L., 2011 A genome-scale shRNA resource for transgenic RNAi in *Drosophila*. Nat. Methods 8: 405–407. 21460824 10.1038/nmeth.1592 PMC3489273

[bib31] Nishihara T. Zekri L. Braun J. E. Izaurralde E., 2013 miRISC recruits decapping factors to miRNA targets to enhance their degradation. Nucleic Acids Res. 41: 8692–8705. 23863838 10.1093/nar/gkt619 PMC3794582

[bib32] Nüsslein-Volhard C. Wieschaus E. Kluding H., 1984 Mutations affecting the pattern of the larval cuticle in *Drosophila melanogaster*. Rouxs Arch. Dev. Biol. 193: 267–282. 10.1007/BF00848156 28305337

[bib33] Nüsslein-Volhard C. Frohnhöfer H.-G. Lehmann R., 1987 Determination of anteroposterior polarity in *Drosophila*. Science 238: 1675–1681. 3686007 10.1126/science.3686007

[bib34] Page S. L. McKim K. S. Deneen B. van Hook T. L. Hawley R. S., 2000 Genetic studies of *mei-P26* reveal a link between the processes that control germ cell proliferation in both sexes and those that control meiotic exchange in *Drosophila*. Genetics 155: 1757–1772. 10924472 10.1093/genetics/155.4.1757 PMC1461182

[bib35] Parisi M. Lin H., 1999 The *Drosophila pumilio* gene encodes two functional protein isoforms that play multiple roles in germline development, gonadogenesis, oogenesis and embryogenesis. Genetics 153: 235–250. 10471709 10.1093/genetics/153.1.235 PMC1460748

[bib36] Perrimon N. Gans M., 1983 Clonal analysis of the tissue specificity of recessive female sterile mutations of *Drosophila melanogaster* using a dominant female sterile mutation *Fs(1)K1237*. Dev. Biol. 100: 365–373. 6418585 10.1016/0012-1606(83)90231-2

[bib37] Perrimon N. Engstrom L. Mahowald A. P., 1984 The effects of zygotic lethal mutations on female germ-line functions in *Drosophila*. Dev. Biol. 105: 404–414. 6479445 10.1016/0012-1606(84)90297-5

[bib38] Perrimon N. Engstrom L. Mahowald A. P., 1989 Zygotic lethals with specific maternal effect phenotypes in *Drosophila melanogaster*. I. Loci on the X chromosome. Genetics 121: 333–352. 2499512 10.1093/genetics/121.2.333 PMC1203621

[bib39] Petrella L. N. Smith-Leiker T. Cooley L., 2007 The Ovhts polyprotein is cleaved to produce fusome and ring canal proteins required for *Drosophila* oogenesis. Development 134: 703–712. 17215303 10.1242/dev.02766

[bib40] Pradhan S. J. Nesler K. R. Rosen S. F. Kato Y. Nakamura A., 2012 The conserved P body component HPat/Pat1 negatively regulates synaptic terminal growth at the larval *Drosophila* neuromuscular junction. J. Cell Sci. 125: 6105–6116. 23097047 10.1242/jcs.113043 PMC3585522

[bib41] Robertson S. E. Dockendorff T. C. Leatherman J. L. Faulkner D. L. Jongens T. A., 1999 *germ cell-less* is required only during the establishment of the germ cell lineage of *Drosophila* and has activities which are dependent and independent of its localization to the nuclear envelope. Dev. Biol. 215: 288–297. 10545238 10.1006/dbio.1999.9453

[bib42] Roth S. Neuman-Silberberg F. S. Barcelo G. Schüpbach T., 1995 *cornichon* and the EGF receptor signalling process are necessary for both anterior-posterior and dorsal-ventral pattern formation in *Drosophila*. Cell 81: 967–978. 7540118 10.1016/0092-8674(95)90016-0

[bib43] Rouget C. Papin C. Boureux A. Meunier A. C. Franco B., 2010 Maternal mRNA deadenylation and decay by the piRNA pathway in the early *Drosophila* embryo. Nature 467: 1128–1132. 20953170 10.1038/nature09465 PMC4505748

[bib44] Sato K. Hayashi Y. Ninomiya Y. Shigenobu S. Arita K., 2007 Maternal Nanos represses *hid/skl*-dependent apoptosis to maintain the germ line in *Drosophila* embryos. Proc. Natl. Acad. Sci. USA 104: 7455–7460. 17449640 10.1073/pnas.0610052104 PMC1854842

[bib45] Schüpbach T. Wieschaus E., 1986 Maternal-effect mutations altering the anterior-posterior pattern of the *Drosophila* embryo. Rouxs Arch. Dev. Biol. 195: 302–317. 10.1007/BF00376063 28306055

[bib46] Schüpbach T. Wieschaus E., 1989 Female sterile mutations on the second chromosome of *Drosophila melanogaster*. I. Maternal effect mutations. Genetics 121: 101–117. 2492966 10.1093/genetics/121.1.101 PMC1203592

[bib47] Schüpbach T. Wieschaus E., 1991 Female sterile mutations on the second chromosome of *Drosophila melanogaster*. II. Mutations blocking oogenesis or altering egg morphology. Genetics 129: 1119–1136. 1783295 10.1093/genetics/129.4.1119 PMC1204776

[bib48] Syrjänen L. Tolvanen M. E. Hilvo M. Vullo D. Carta F., 2013 Characterization, bioinformatic analysis and dithiocarbamate inhibition studies of two new α-carbonic anhydrases, CAH1 and CAH2, from the fruit fly *Drosophila melanogaster*. Bioorg. Med. Chem. 21: 1516–1521. 22989910 10.1016/j.bmc.2012.08.046

[bib49] Tadros W. Goldman A. L. Babak T. Menzies F. Vardy L., 2007 SMAUG is a major regulator of maternal mRNA destabilization in *Drosophila* and its translation is activated by the PAN GU kinase. Dev. Cell 12: 143–155. 17199047 10.1016/j.devcel.2006.10.005

[bib50] Wehr K. Swan A. Schüpbach T., 2006 Deadlock, a novel protein of *Drosophila*, is required for germline maintenance, fusome morphogenesis and axial patterning in oogenesis and associates with centrosomes in the early embryo. Dev. Biol. 294: 406–417. 16616913 10.1016/j.ydbio.2006.03.002

[bib51] Zaessinger S. Busseau I. Simonelig M., 2006 Oskar allows *nanos* mRNA translation in *Drosophila* embryos by preventing its deadenylation by Smaug/CCR4. Development 133: 4573–4583. 17050620 10.1242/dev.02649

